# Assessing mutation accumulation in DNA repair-deficient *Listeria monocytogenes*: implications for cgMLST cluster thresholds in outbreak analysis

**DOI:** 10.3389/fcimb.2025.1530851

**Published:** 2025-02-17

**Authors:** Astrid Füszl, Ariane Pietzka, Patrick Hyden, Tobias Mösenbacher, Anna Stöger, Marion Blaschitz, Silke Stadlbauer, Petra Hasenberger, Stefanie Schindler, Florian Heger, Sonja Pleininger, Alexander Indra

**Affiliations:** ^1^ National Reference Centre/Laboratory for Listeriosis, Austrian Agency for Health and Food Safety (AGES), Vienna/Graz, Austria; ^2^ Department of Statistics and Analytical Epidemiology, Austrian Agency for Health and Food Safety (AGES), Vienna, Austria; ^3^ Department of Clinical Molecular Biology, Austrian Agency for Health and Food Safety (AGES), Vienna, Austria

**Keywords:** *Listeria monocytogenes*, listeriosis, mutation rate, core genome multilocus sequence typing, allele threshold, outbreak detection

## Abstract

**Background:**

*Listeria (L.) monocytogenes* is primarily transmitted via contaminated food and can cause listeriosis, an infection often associated with sepsis and meningitis in at-risk individuals. Accurate outbreak detection relies on whole genome sequencing (WGS) and core genome multilocus sequence typing (cgMLST), which use allele thresholds to identify related strains.

**Methods:**

This study investigated mutation rates in *L. monocytogenes*, focusing on isolates with DNA repair deficiencies. Serial subcultivations were performed, comparing a repair-deficient isolate with a wild-type control. Genetic variability was assessed using WGS and cgMLST.

**Results:**

Mutation rates were significantly higher in repair-deficient isolates, exceeding typical cgMLST thresholds currently used in *Listeria* outbreak investigations, leading to a misclassification of related isolates as unrelated. An additional analysis of the Austrian *Listeria* database revealed that such deficiencies are rare among isolates.

**Conclusions:**

The standard 7-allele cgMLST threshold effectively identifies related strains in most cases, but may require adjustments for hypermutator strains. Incorporating DNA repair data could improve the accuracy of outbreak investigations, ensuring reliable public health responses.

## 1 Introduction

Listeriosis, caused by the bacterium *L. monocytogenes*, is a serious foodborne illness with considerable public health impact, particularly due to its severe outcomes in high-risk groups. Vulnerable groups - including the elderly, pregnant women, newborns, and immunocompromised individuals - are especially susceptible to invasive forms of the disease, such as bacteremia and meningitis. These conditions are associated with high case-fatality rates, highlighting the importance of robust food safety and surveillance systems to promptly detect and manage outbreaks by rapidly identifying contaminated food sources. Recent data from the European Centre for Disease Prevention and Control (ECDC) indicate a rise in listeriosis cases across Europe, underscoring the ongoing challenge this infection poses to public health (European Centre for Disease Prevention and Control, 2023).

In Austria, the submission of *L. monocytogenes* isolates from human, food, and environmental sources to the National Reference Laboratory (NRL) has been mandatory since 2014. Since 2016, these isolates have been routinely analyzed using whole genome sequencing (WGS) and core genome multilocus sequence typing (cgMLST) (Cabal et al., 2019; Pietzka et al., 2019). The NRL manages WGS data in a central database, applying cgMLST to track clusters and trace potential contamination sources. This systematic monitoring aligns with an EU-wide initiative that mandates the notification of invasive listeriosis cases and uses WGS-based surveillance as a cornerstone for early outbreak detection and control.

In *Listeria* outbreak investigations, cgMLST is a widely utilized technique with high discriminatory power. It enables the identification of genetically related clones by analyzing conserved genes within the *L. monocytogenes* genome. For typing of L. monocytogenes, the Ruppitsch et al (European Centre for Disease Prevention and Control, 2020). scheme with 1,701 target genes as well as the Pasteur scheme by Moura et al (Ruppitsch et al., 2015). with 1,748 target genes are commonly used cgMLST schemes, playing a crucial role in the harmonization of surveillance efforts across EU member states (Ruppitsch et al., 2015; Moura et al., 2016; European Centre for Disease Prevention and Control, 2020).

An essential aspect of cgMLST in *Listeria* outbreak detection is the application of a cluster threshold - typically 7-10 allele differences - to separate outbreak-related cases from sporadic ones. This threshold is grounded in research showing that isolates from the same outbreak generally differ by fewer than 7-10 alleles (Ruppitsch et al., 2015; European Centre for Disease Prevention and Control, 2022). By applying this limit, investigators can effectively determine when to initiate outbreak investigations and optimize food traceback efforts.

The integration of molecular data with epidemiological evidence, including patients’ food histories, has proven essential in several outbreak investigations, enabling the identification of contaminated food sources and the swift implementation of control measures, such as food product recalls. For instance, a *L. monocytogenes* outbreak involving 22 cases across five EU member states from 2014 to 2019 was linked to contaminated salmon products using cgMLST (European Centre for Disease Prevention and Control and European Food Safety Authority, 2019). Similarly, in Andalusia, Spain, an outbreak from July to October 2019 involving 207 confirmed listeriosis cases was linked to contaminated stuffed pork (Fernández-Martínez et al., 2022).

Although the widely used 7-allele cgMLST threshold is generally effective for assessing *L. monocytogenes* isolate relatedness, it may not be universally applicable. Isolates with DNA repair deficiencies, for example, can exhibit accelerated mutation rates, resulting in higher allele differences despite originating from the same strain (Mérino et al., 2002; Koomen et al., 2018; Füszl et al., 2024). Our study builds on previous findings of substantial genetic divergence due to DNA repair deficiencies, notably in a chronic listeriosis case where a patient experienced six bacteremic episodes. In this case, one of the isolates exhibited a 20-allele difference in cgMLST, linked to mutations in genes critical for DNA repair (Füszl et al., 2024).

This follow-up study tested the hypothesis that DNA repair-deficient isolates accumulate mutations more rapidly. To investigate this, serial subcultivations of the patient’s defective isolate were performed under various conditions, and its mutation rates were compared to those of a non-defective isolate from the same patient. The aim was to assess how these mutation dynamics might affect the reliability of the cgMLST threshold currently used in *Listeria* outbreak investigations. Additionally, the prevalence of DNA repair defects in *Listeria* was evaluated by analyzing all *Listeria* sequences currently available in the Austrian database.

## 2 Materials and methods

### 2.1 Preliminary findings from previous research

#### 2.1.1 Patient isolate collection and initial cgMLST analysis

This study builds on earlier research involving a patient with chronic listeriosis. Over a two-year period, six isolates were collected during separate episodes of bacteremia, all traced back to a colonized implantable cardioverter-defibrillator (ICD) device (Füszl et al., 2024). The cgMLST analysis was performed using the cgMLST scheme developed by Ruppitsch et al (Ruppitsch et al., 2015). One isolate exhibited significant genetic divergence, showing a 20-allele difference from the patient’s initial isolate. This finding suggested either a reinfection with a different strain or a relapse caused by the emergence of a distinct sublineage within the same strain.

#### 2.1.2 Identification of DNA repair defects and implications

Subsequent genetic analyses uncovered mutations in two key genes involved in DNA repair: the *mutS* gene, encoding a DNA mismatch repair protein that detects and repairs errors in DNA replication, and the *mutS-2* gene, encoding a protein likely acting as a recombination and DNA strand exchange inhibitor protein. These mutations likely compromised the isolate’s ability to effectively repair DNA defects, leading to an accumulation of genetic mutations over time. This discovery highlighted the need for further investigations into how such DNA repair deficiencies might contribute to the genetic divergence observed in cgMLST analyses.

### 2.2 Serial subcultivation

To evaluate the effect of defective DNA repair mechanisms on genetic variability, the patient isolate with the DNA repair defect (= mutant isolate, MI) was subjected to serial subcultivations. For comparison, a control isolate obtained from the same patient, but lacking these DNA repair defects (= wild-type isolate, WT), was also subcultured under identical conditions. This approach aimed to quantify and compare mutation rates between the defective and wild-type isolates.

Specifically, both isolates were cultured on Columbia Agar supplemented with 5% sheep blood (BioMérieux, Marcy-l’Étoile, France):

- Aerobic incubation at 37°C for 48 hours
- Aerobic incubation at 4-8°C for 48 hours (refrigerated conditions)
- Aerobic incubation at 37°C for 48 hours with the addition of penicillin discs (antibiotic stress)

Different conditions were selected to simulate potential stressors that *L. monocytogenes* might encounter in a food or host environment, including temperature fluctuations and antibiotic exposure. From each of the six plates (two isolates × three conditions), subcultures were performed every two days, resulting in a total of 14 subcultures.

It was hypothesized that more frequent subculturing would lead to greater mutation accumulation, particularly in the DNA repair-deficient isolate, due to increased replication errors. This experimental design was intended to evaluate the effects of subculturing frequency and environmental stressors on mutation rates.

### 2.3 cgMLST analysis

Subsequently, WGS was performed on colonies from various subcultures. Specifically, isolates were sequenced starting with subculture 4, and then from every second subculture thereafter (subcultures 4, 6, 8, 10, 12, 14), with a single colony selected per plate.

Genomic DNA was extracted using the MagAttract HMW DNA Kit (QIAGEN, Hilden, Germany) according to manufacturer’s instructions. DNA concentration was determined with the Dropsense 16 (Unchained Labs, USA). For library preparation, the Nextera XT DNA Library Prep Kit (Illumina, San Diego, CA, USA) was used. Sequencing was performed on a NextSeq 2000 (Illumina) with v3 chemistry, generating 150 base pair paired-end reads. The FASTQ files obtained were post-processed with Trimmomatic (parameters: SLIDINGWINDOW:30:20) to improve read quality and *de novo* assembled using SPAdes 3.15.5.

Core genome multilocus sequence typing (cgMLST) was conducted using Ridom SeqSphere+ software version 10.0.4 (Ridom, Münster, Germany), as described by Ruppitsch et al (Ruppitsch et al., 2015). Allele differences were compared across successive generations. Missing values were pairwise ignored, and isolate relatedness was visualized using a minimum spanning tree (MST) generated with Ridom SeqSphere+.

### 2.4 In-depth genomic analysis

A mutation analysis was performed using Snippy (v=4.6.0) with parameters: min-frac 0.7 and min-coverage 5 to detect single nucleotide polymorphisms (SNPs), insertions and deletions, using the hybrid assembly of the original WT isolate from the study by Füszl et al. as a reference (Füszl et al., 2024).

The detected SNPs and indels were treated as categorical values (“profiles”) and used to generate an MST visualization with grapetree v.1.5.0 using default parameters (Zhou et al., 2018) (**Supplementary Datasheet 1**).

Genomic rearrangements were analyzed by creating scaffolds from short-read *de novo* assemblies using the hybrid assemblies from Füszl et al. as a reference in RagTag (Alonge et al., 2019; Füszl et al., 2024). The largest scaffolds per sample (“pseudo-chromosome”) were compared using SyRI (Goel et al., 2019).

### 2.5 Listeria database investigation

In Austria, all isolates of *L. monocytogenes* must be submitted to the NRL for typing, irrespective of their source. Sources include clinical isolates as well as food-associated and environmental isolates from official controls and own checks. All isolates are routinely typed using cgMLST analysis based on the method established by Ruppitsch et al. (Ruppitsch et al., 2015). and are stored in a Ridom SeqSphere+ database, which currently contains over 22,000 sequences (as of September 3^rd^, 2024) and is expanded by approximately 3,000 sequences annually.

To assess the frequency of DNA repair defects across a broad range of *Listeria* strains, all isolates from the Austrian strain collection were investigated. This analysis was crucial for understanding the prevalence of DNA repair defects and their potential impact on cgMLST-based outbreak investigations Ruppitsch et al. (Ruppitsch et al., 2015).

## 3 Results

### 3.1 WGS and data analysis

Assembled average coverages for all WGS samples were higher than 50-fold, ranging from 53 to 113-fold. The proportion of targets in the *L. monocytogenes* core genome as defined by Ruppitsch et al. (Ruppitsch et al., 2015) that met quality standards was consistently above 98.5%, ranging from 98.7% to 99.8%. Contig counts varied between 36 and 214.

### 3.2 Mutation rates in serial subcultivation

**Figure 1:**
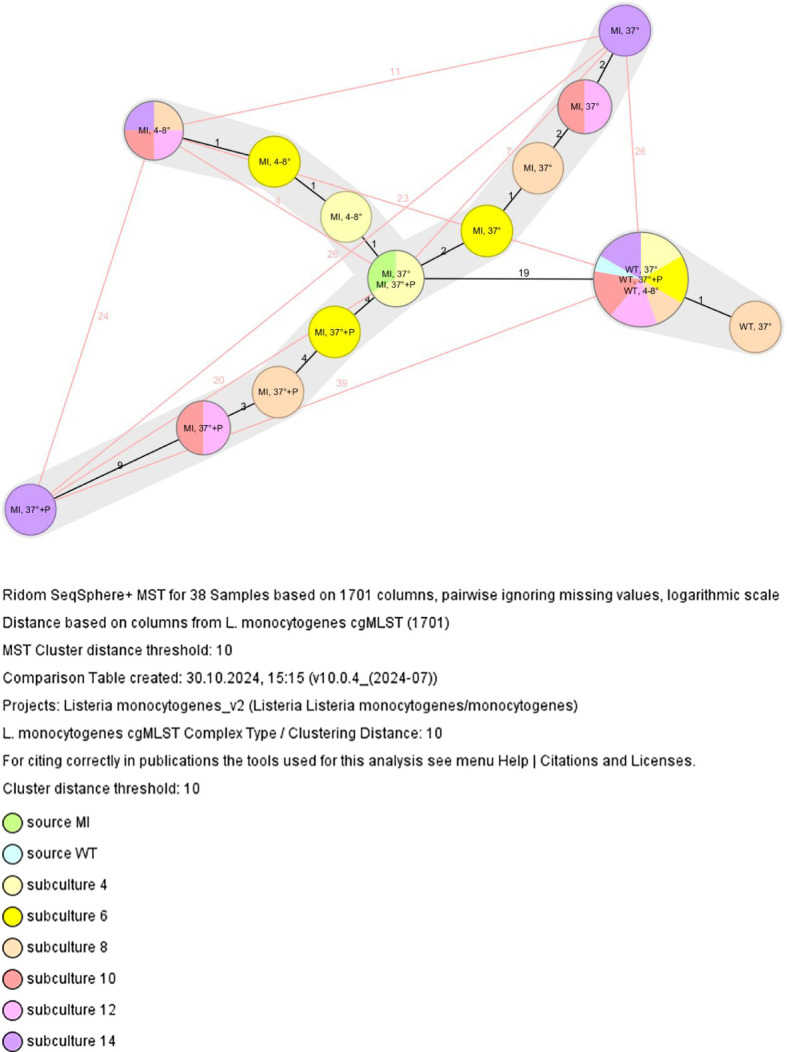
Minimum spanning tree based on the cgMLST allelic profiles of subcultures 4, 6, 8, 10, 12 and 14 of the wild-type and mutant isolate. Each circle represents a given allelic profile. The number on the connecting lines illustrates the number of differing alleles; MI, mutant isolate, WT, wild-type isolate, 37°C: aerobic incubation at 37°C for 48 hours, 4-8°C: aerobic incubation at 4-8°C for 48 hours, 37°C + P: aerobic incubation at 37°C for 48 hours with the addition of penicillin discs.

Subcultivation experiments demonstrated that the DNA repair-deficient isolate (= mutant isolate, MI) accumulated mutations at a markedly higher rate compared to the control isolate (= wild-type isolate, WT). Prior to subculturing, WT (**Figure 1**, light blue) and MI (**Figure 1**, light green) sources differed by 19 alleles, indicating distinct clonal clusters. However, as previously noted by Füszl et al (Füszl et al., 2024), WT and MI originated from the same clone, with MI representing a DNA repair-deficient sublineage of the original WT isolate that had surpassed the defined core genome cluster threshold of 10 alleles per due to its repair deficiencies Ruppitsch et al. (Ruppitsch et al., 2015).

Over time, subculturing of the MI led to a significant increase in allelic differences, whereas WT subculturing had minimal effect, with only one WT subculture (subculture 8) differing by one allele from all other subculture WT isolates (**Figure 1**).

After fourteen rounds of subcultures, allele differences exceeded the cluster threshold, both among MI isolates (under different incubation conditions) and between WT and MI (**Table 1**, **Figure 1**).

**Table 1:** Allelic differences by incubation condition and isolate type.

Isolate type and incubation condition	Allelic difference
MI 37°C – MI 37°C + P	26
MI 37°C – MI 4-8°C	11
MI 4-8°C – MI 37°C + P	24
WT – MI 37°C	26
WT – MI 37°C + P	39
WT – MI 4-8°C	23

MI, mutant isolate; WT, wild-type isolate; 37°C, aerobic incubation at 37°C for 48 hours, 4-8°C, aerobic incubation at 4-8°C for 48 hours, 37°C + P, aerobic incubation at 37°C for 48 hours with the addition of penicillin discs.

**Figure 1** illustrates that incubation of the MI at 37°C with antibiotic exposure had the highest effect on allelic difference (20 alleles) between the MI source and MI subculture 14. Incubation at 4-8°C resulted in the lowest mutation rate, leading to the smallest allelic difference (4 alleles). Incubation at 37°C resulted in a 7-allele difference between the MI source and MI subculture 14. Additionally, WT and MI subcultures 14 showed allele differences of 23, 26 and 39 alleles, respectively.

### 3.3 In-depth genomic analysis findings

The mutation analysis revealed a significantly increased mutation rate in samples with defective repair mechanisms (MI), with an average of 4.94 mutations (SNPs or indels) per sample and cultivation step compared to 0.33 in control samples (WT). The wild-type samples exhibited the following mutation rates per subcultivation step under the different growth conditions: 0.11 mutations at 37°C, 0 mutations at 37°C with penicillin, and 0 mutations at temperatures between 4°C and 8°C. In contrast, the samples with a defective repair mechanism displayed mutation rates of 7.17 mutations at 37°C, 6 mutations at 37°C with penicillin, and 1.67 mutations at temperatures between 4°C and 8°C.

The apparently higher mutation rate observed in MI 37°C + penicillin isolates, compared to MI 37°C subcultivation in core-genome MLST genes, was not confirmed by SNP/indel detection using Snippy over entire genomes.

In total, 35 distinct deletions and 21 distinct insertions were identified compared to the WT source. Notably, these indels were exclusively observed in samples with defective repair mechanisms and consisted solely of homopolymer insertions or deletions leading to frameshifts in 40 predicted coding regions. All identified SNPs were transitions. No mutations were observed to arise during cultivation and subsequently disappear in later cultures. Furthermore, no mutations occurred independently multiple times in different subcultivations.

A comparison between the original wild-type isolate and the original mutant isolate revealed a single genomic rearrangement. However, no additional rearrangements were detected in the reconstructed scaffolds of subcultivated wild-type or mutant isolates.

### 3.4 Prevalence of DNA repair defects

The analysis of the *Listeria* database identified a total of five isolates with similar DNA repair defects among over 22,000 sequences: two isolates had a mutation in the *mutS* gene, and three isolates had a mutation in the *mutS-2* gene. Notably, no isolate exhibited mutations in both genes.

## 4 Discussion

Our study highlights that *L. monocytogenes* isolates with compromised DNA repair mechanisms, such as those with mutations in the *mutS* and *mutS-2* genes, exhibit elevated mutation rates. These higher rates were observed during serial subcultivations, leading to allele differences that challenge the conventional 7-allele cluster threshold currently used to identify related strains in *Listeria* outbreak investigations.

This observation aligns with research by Chopra et al., who highlighted the role of mutator strains, particularly those with *mutS* defects, in driving genetic diversity and the evolution of antibiotic-resistant bacteria (Chopra et al., 2003). Mérino et al. also found that hypermutator strains of *L. monocytogenes* with mutations in *mutL* and *mutS* genes exhibited increased mutation rates but also reduced virulence, suggesting a trade-off between genetic diversity and pathogenicity (Mérino et al., 2002). Similarly, a study on stress exposure as a catalyst for adaptive evolution indicated that mutation rates increased only in *Listeria* strains with DNA repair deficiencies, specifically with a mutation in the *mutS* DNA mismatch repair gene (Koomen et al., 2018). However, even in the absence of antibiotic stress, the mutation rate of our hypermutator strain remained high, as the *mutS* defect is the main driver of the mutation rate in this strain, independent of external stress factors. Refrigeration temperatures as a potential stressor reduced mutation rates in mutant isolates, likely due to *L. monocytogenes’* reduced division rate at lower temperatures, which may limit the overall accumulation of mutations.

To assess how common DNA repair deficiencies are, this study examined the Austrian *Listeria* database, which currently contains over 22,000 sequences. Our analysis found that such repair deficiencies are rare, with only five isolates exhibiting similar defects. In the absence of DNA repair defects, however*, L. monocytogenes* typically exhibits a highly stable genome, with clusters of closely related strains often persisting over extended periods (Jackson et al., 2016). This is attributed to the organism’s low mutation rate of approximately one SNP per year as well as its ability to form resilient biofilms, allowing it to persist in various settings such as food processing environments (Keto-Timonen et al., 2007; Orsi et al., 2008; Moura et al., 2016; Van Walle et al., 2018).

These findings indicate that the current 7-allele cgMLST threshold remains robust for most *L. monocytogenes* outbreak investigations, but may need reconsideration in rare instances of hypermutator phenotypes.

Supporting this, Van Walle et al. propose that a higher cut-off might be required to more accurately differentiate between confirmed and probable outbreak cases (Van Walle et al., 2018). Jackson et al. further noted that a single cut-off may not reliably predict epidemiological relatedness. They argue that isolates with fewer than 10 cgMLST allele differences are often epidemiologically linked, those with 10–30 differences are frequently linked, and isolates with more than 30 differences are occasionally linked (Jackson et al., 2016).

Given the critical role of accurately establishing microbiological relatedness for public health, the integration of WGS into routine surveillance has undoubtedly enhanced *L. monocytogenes* outbreak detection and response, enabling precise tracking across different sources and regions. However, maintaining this accuracy in the face of genetic anomalies like DNA repair deficiencies will require defining when a higher allele cut-off or inclusion of gene-level information – such as DNA repair status – might be appropriate. This approach would help avoid misclassifying genetically related isolates as unrelated, potentially hampering or delaying outbreak detection and control measures.

In conclusion, although the 7-allele threshold remains effective for most *L. monocytogenes* outbreak investigations, rare instances of hypermutator strains with DNA repair deficiencies pose unique challenges, potentially requiring adjusted thresholds. Incorporating additional genomic data, such as DNA repair status, into cgMLST interpretation will enhance the accuracy and reliability of outbreak investigations, ensuring that public health responses remain both timely and effective.

## Data Availability

The datasets presented in this study can be found in online repositories. The names of the repository/repositories and accession number(s) can be found below: https://www.ebi.ac.uk/ena, PRJEB83895.
