# Supplementary material for: Assessing mutation accumulation in DNA repair-deficient Listeria monocytogenes: implications for cgMLST cluster thresholds in outbreak analysis
Source: Front Cell Infect Microbiol. 2025 Feb 17;15:1530851. doi: 10.3389/fcimb.2025.1530851 (PMC11872914; doi:10.3389/fcimb.2025.1530851)
Supplement: Supplementary file 1 [file DataSheet1.pdf]

Supplementary Figure 1.

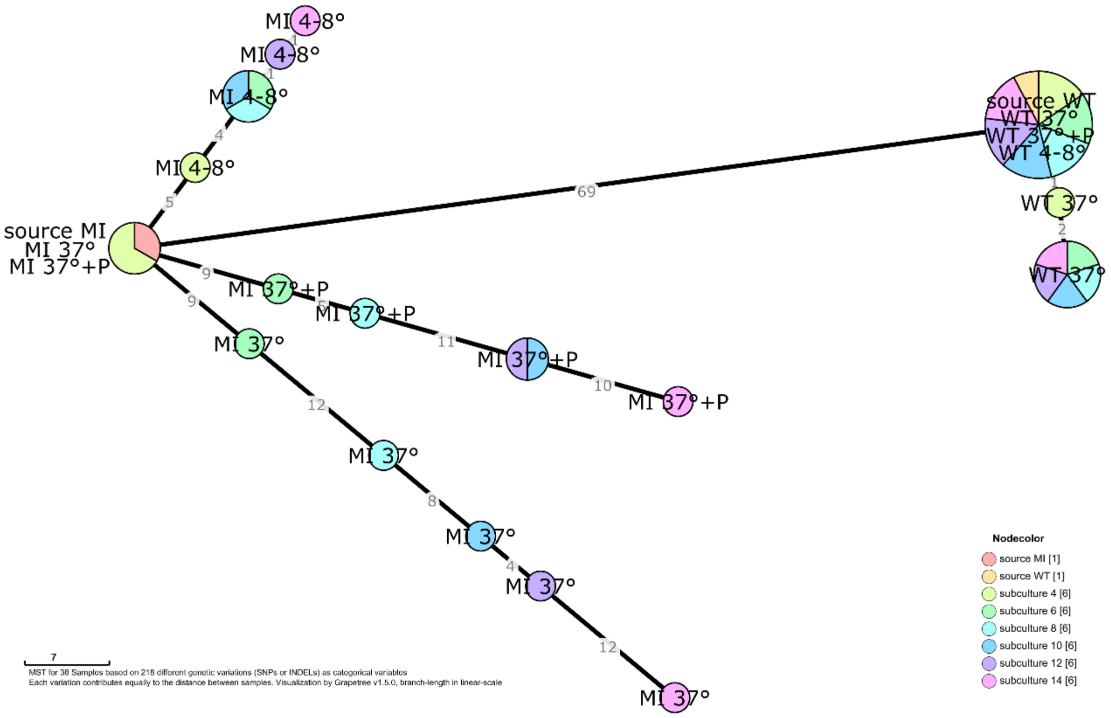

Supplementary Figure 1. A minimum spanning tree (MST) was created using grapetree v.1.5.0. Mutations (SNPs and indels) were used as categorical profiles for grapetree to generate an unrooted tree. MI: mutant isolate, WT: wild-type isolate, 37 °C: aerobic incubation at 37 °C for 48 hours, 4-8 °C: aerobic incubation at 4-8 °C for 48 hours, 37 °C + P: aerobic incubation at 37 °C for 48 hours with the addition of penicillin discs. The subcultivations of the wild-type strain (WT) exhibited fewer genetic changes over time compared to the subcultivations of the repair-deficient mutant strain (MI).
